# [Retracted] MicroRNA-25-3p regulates human nucleus pulposus cell proliferation and apoptosis in intervertebral disc degeneration by targeting Bim

**DOI:** 10.3892/mmr.2026.13845

**Published:** 2026-03-16

**Authors:** Zhifang Zhao, Jie Zheng, Youchen Ye, Kefeng Zhao, Ruozhang Wang, Ran Wang

Mol Med Rep 22: 3621–3628, 2020; DOI: 10.3892/mmr.2020.11483

Following the publication of the above paper, it was drawn to the Editor's attention by a concerned reader that certain of the flow cytometric data shown in Figs. 3D and 5D on p. 3625 and p. 3626 respectively were strikingly similar to data that had appeared previously in other papers written by different authors at different research institutes, which had already been accepted for publication.

In view of the fact that the abovementioned data had already apparently been published prior to its submission to *Molecular Medicine Reports*, the Editor has decided that this paper should be retracted from the Journal. The authors were asked for an explanation to account for these concerns, but the Editorial Office did not receive a reply. The Editor apologizes to the readership for any inconvenience caused.

